# Position-Dependent Intrathecal Baclofen System Catheter Failure Resulting in Debilitating Spasticity: A Case Report

**DOI:** 10.7759/cureus.53425

**Published:** 2024-02-01

**Authors:** Elvis Guzman, Cody Barbari, Joseph Paganoni, Jackson Cohen, Joanne Delgado-Lebron

**Affiliations:** 1 Physical Medicine and Rehabilitation, Memorial Healthcare System, Hollywood, USA; 2 Medical School, American University of the Caribbean School of Medicine, Cupecoy, MAF

**Keywords:** intrathecal drug delivery, pain, withdrawal, catheter, spasticity, baclofen

## Abstract

An intrathecal baclofen pump (ITB) can provide significant relief from excessive spasticity and pain that is difficult to control. However, it is not without its drawbacks. We present a case of a young quadriplegic male who underwent ITB pump placement, suffering four years of transient episodes of severe spasticity with withdrawal symptoms. Multiple adjustments were made to his ITB pump dosing without relief. Extensive workup including interrogation of the pump, serial abdominal radiographs, and fluoroscopic catheter dye study revealed no abnormalities. Intraoperatively, it was discovered that the initial catheter anchoring occurred directly adjacent to the vertebrae, leading to a position-dependent catheter occlusion. He underwent the replacement of his ITB pump and catheter. During surgical revision, emphasis was placed on reducing the length of the catheter outside the spine, anchoring to the supraspinous fascia with avoidance of bony prominences or post-laminectomy sites. After surgery, the patient's spasticity improved, and at the eight-month follow-up, he had no complications, resulting in a mean baclofen dose of 300.2 μg/day. This report highlights the potential risk of life-threatening intrathecal baclofen withdrawal secondary to postural changes, providing technical considerations to prevent recurrences. It also raises awareness regarding patients who are more susceptible to transient catheter occlusion after a spinal cord injury.

## Introduction

Although the exact prevalence is unknown, it is estimated that more than 12 million people worldwide may suffer from spasticity [1] and that 12-27% of them, depending on the etiology, have disabling spasticity [2]. There is considerable evidence that spasticity negatively affects patients by causing physical disability, activity limitations, dependence on healthcare providers, limited participation in family and social life, and reduced overall quality of life [3,4]. 

Individuals with conditions such as cerebral palsy, spinal cord injuries, traumatic brain injuries, and multiple sclerosis may experience severe and uncontrollable muscle stiffness and rigidity, which can result in substantial impairment. Most patients experience success with oral antispasmodic therapy using baclofen. However, a considerable number of patients may not achieve positive outcomes due to the severity of their symptoms or the undesirable side effects. In these circumstances, administering baclofen through a surgically implanted infusion pump directly into the spinal cord offers a stronger muscle relaxant effect with fewer adverse reactions [5].

Positive findings regarding the management of spasticity with intrathecal baclofen (ITB) pumps have been documented [6], although there have been instances of mechanical and surgical complications. These complications can include infection, drug leakage, hardware failure, or drug side effects [7,8]. Earlier research has indicated that a considerable proportion of patients (up to 40%) have experienced issues such as catheter dislodgment, kinking, tearing, and disconnection [9]. Understanding and addressing these potential complications is critical for patients considering or undergoing baclofen pump placement, as well as for the healthcare professionals involved in their care. However, detecting the underlying issue might prove to be challenging.

In this case, we present a patient who experienced transient occlusion of the catheter resulting in a failure to administer ITB leading to a resurgence of his pain and spasticity, a year after ITB pump placement.

## Case presentation

A 24-year-old male with a history of chronic C5 incomplete quadriplegia complicated by severe generalized spasticity was being followed in our clinic for ITB pump management five years after implantation of the pump. Anthropometric data were collected which included height 182.9 cm, weight 60 kg, and BMI 17.94 kg/m². The patient underwent ITB pump placement one year post injury, later complicated by a dislodged catheter requiring replacement of the catheter done through an L3-L4 laminectomy with catheter advanced up to T11 level. It is entirely unclear as to why laminectomy was needed for the ITP catheter revision as this was done at an outside facility with documentation not available to corroborate. Approximately one year after this revision, the patient started experiencing transient episodes of severe spasticity involving bilateral lower extremities with withdrawal symptoms including itchiness. There were concerns for intermittent withdrawal due to a possible kinked catheter, as episodes occurred either when lying prone or with back extension positioning, such as when using a foam roller while supine. The resulting spasticity required an additional 30-40 mg of oral Baclofen every four hours in addition to his programmed pump dosing of 289.1 μg/day for withdrawal abatement. These episodes were followed by days of total flaccid tone in bilateral lower extremities. 

**Figure 1:**
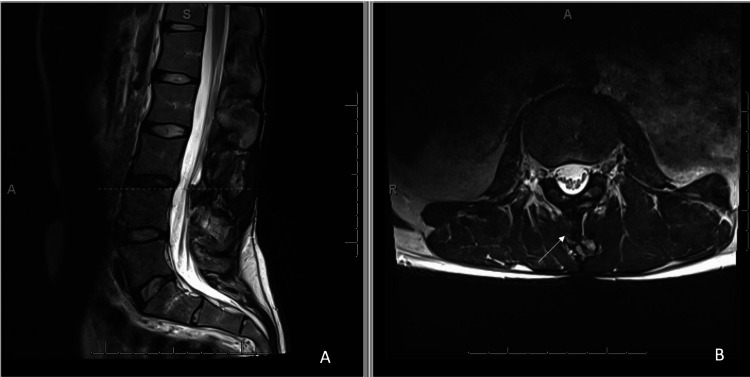
MRI lumbar spine without contrast, sagittal T2 and axial T2 at L3-L4 level (A) L3-L4 post-laminectomy changes with disc desiccation at the L3-L4 vertebral body level (dashed line); (B) Cross-section of post-laminectomy site with calcifications in and around the interlaminar space (arrow).

A catheter dye study was performed at which time the catheter position was confirmed to be appropriate with clear fluid aspirated from the port, and no evidence of a kink or obstruction was found. Pump interrogation was done with no apparent problems. He was recommended to avoid any back extension activity during dose titration. At that time, his symptoms of rebound spasticity were presumed to be related to decreasing the dosage of his baclofen pump infusion. Dosage was increased by 10%, back to the previous dosage, with low suspicion of catheter malfunction given negative dye study previously. Meanwhile, despite this increase in the dose of ITB, the patient's spasticity worsened, necessitating a visit to the emergency department. An investigation was performed to determine the underlying etiology of his spasticity including MRI lumbar spine (Figure 1), complete blood count, urinalysis, urine culture, and abdominal radiograph to evaluate for urinary tract infection (UTI) or constipation.

The patient was diagnosed with a UTI and discharged home on antibiotics. On the second day of antimicrobial therapy, the patient reported that after lower extremity passive range of motion exercises, he awoke the next morning with completely flaccid tone of his legs and very minimal spasms when he would transfer. The patient was seen in the clinic the following day with a notable decrease in lower extremity tone, beyond what can be seen by the third day of antibiotic treatment for UTI. Although UTIs are known to trigger spasticity, consideration was made towards catheter malfunction that could be positional. 

**Figure 2:**
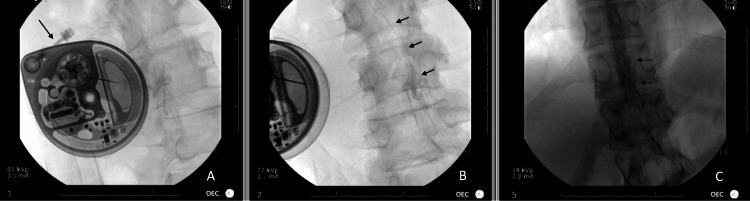
Fluoroscopic catheter dye study (A) Fluoroscopic lateral view with needle (arrow) in the side port with no extravasation of contrast around the pump; (B) Fluoroscopic oblique view with no extravasation from the catheter (arrows); (C) Catheter tip at the T5 level with a gravity-dependent spread of contrast into the thecal space (arrows).

Throughout his follow-up appointments, he continued to consistently report withdrawal symptoms after each dose adjustment. Serial abdominal radiographs were negative for a dislodged catheter. The patient was referred to an interventional pain specialist for a repeat catheter dye study. The fluoroscopic catheter dye study revealed no abnormalities with the catheter or pump (Figure 2).

Based on the patient's history of episodes of completely uncontrollable spasticity with withdrawal symptoms followed by days of complete flaccidity, this again suggested a possible transient catheter problem rather than a failed pump. It was recommended to replace the entire system, considering the patient had been dealing with this problem for several years and it was affecting his quality of life. The patient was advised to continue taking oral baclofen to avoid early withdrawal symptoms if they persisted. 

The patient underwent a replacement of his ITB pump and catheter. With the use of fluoroscopy, we identified the L3-L4 interlaminar spaces and noted the location of the previous anchor. A vertical incision was then made with a scalpel followed by electrocautery to dissect down to the thoracolumbar fascia layer. We made the incision slightly to the right of midline. Once the lumbar fascia layer was visualized, we noted the catheter coming through the fascia at around the L3-L4 level. We palpated the anchor deeper, adjacent to the spinous process below the muscle layer. Once we had the fascia exposed, we then identified the interlaminar spaces. We first attempted at the L2-L3 interlaminar space with the spinal needle, but we were unable to enter the space due to calcifications in and around the interlaminar space. We then directed the needle towards the L1-L2 interlaminar space and reconfirmed on MRI that the spinal cord ended at the L1 vertebral body level and at the L1-L2 interlaminar space the canal was open with only cauda equina nerve roots.

We used a paramedian oblique entry into the L1-2 interlaminar space on the right side. A lateral view then confirmed the depth of the needle and the needle was advanced parallel to the dural fibers into the intrathecal space. The stylet from the needle was then removed slightly and clear cerebrospinal fluid (CSF) was seen to be flowing. Next, the intrathecal catheter was introduced into the needle and advanced in the intrathecal space using anterioposterior and lateral fluoroscopy to confirm location up to the T5 vertebral body level. Then, two anchoring sutures were placed using 0 Ethibond (Johnson & Johnson MedTech, New Jersey, United States) on either side of the needle into the fascia. The needle and stylet from the catheter were then removed maintaining the intrathecal catheter at the T5 level checked on fluoroscopy. CSF was seen to be coming out of the tip of the catheter confirming intrathecal location. The tip of the catheter was then clamped to the drape. The catheter was then unclamped, and the anchor placed over the catheter into the fascia with the wings touching the fascia on either side. The tip of the catheter was then clamped again. Next, the 0 Ethibond sutures were tied into the holes of the wing of each side of the anchor securing them into the fascia, away from bony prominences. The old pump was removed and the old catheter was cut at the lumbar incision site and pulled through the tunnel out through the abdominal pocket intact. The abdominal pocket was then irrigated with antibiotic irrigation and hemostasis was obtained. The new catheter was then coiled underneath the pump and the new 20 mL pump was placed into the abdominal pocket. 

Following the procedure, his spasticity has been much better controlled. The patient reports that spasticity occurred only in the morning with bed mobility. The patient denies baclofen withdrawal episodes such as those experienced previously. At this point, the patient was very happy with the results of their pump, receiving a simple continuous infusion at 300.2  μg/day. 

## Discussion

The ITB pump is intended for administering lesser amounts of baclofen directly into the subarachnoid space. While this method of treatment can provide significant relief for excessive spasticity and pain that is difficult to manage, it is not without its drawbacks. Simply put, complications can be categorized as either related to the surgical implantation and mechanical functioning of the infusion device or to the drug itself. Regarding the role of the infusion device, the catheter is identified as the most susceptible component within the system [10], with complications previously reported reaching 31-35% [11,12]. More than half of the issues with catheters were caused by bending, breaking, or tearing of the slender elastic catheter. Nevertheless, the prevalence of this issue has significantly decreased to 1.1% with the utilization of the newly developed Ascenda catheter (Medtronic plc, Dublin, Ireland) [13]. 

When it comes to the possibility of catheter failure, the main reason for such a failure is a migration of the catheter, which occurs when it fails to properly secure itself onto the surrounding fascia. As suggested by Follett et al., this could be due to the assumption that the subsequent scar tissue or suture would be sufficient to secure the catheter [14]. On the other hand, preventing the catheter from kinking, particularly when anchored directly adjacent to the vertebrae, is another important consideration, especially since transient obstruction can result during bed mobility and transfers. Although the technique used in surgery is relevant, the construction of the catheter (whether it is one-piece or two-piece, its thickness, and the materials used during production) is also believed to have a considerable influence. Common problems with two-piece designs are the occurrence of kinking, disconnection, and dislodgement. The multiple lateral holes along the tip, rather than a single distal opening, may reduce the likelihood of granuloma formation at the tip end allowing for a wider distribution of the drug through the cerebrospinal fluid [14]. Additionally, the potential role of interlaminar calcifications surrounding the catheter in causing temporary obstruction must also be considered. Moreover, individuals with substantial disabilities who have ITB pumps often experience extended periods of inactivity. During these periods, one could be resting in a supine position. If the catheter is located near the neighboring vertebrae, prolonged compression can result in the bending of the catheter and increase the chance of deterioration. Eventually, this leads to damage and leakage. 

There are certain measures that can be followed during the surgical insertion of the device to minimize the frequency of complications mentioned up to this point. To start, it is recommended to reduce the length of the catheter outside the spine since there is a higher risk of it bending and breaking. To correctly position and prevent the catheter from moving unnecessarily, it is recommended to secure it via a dedicated butterfly anchor to the supraspinous fascia, away from bony prominences. In our situation, the previous anchor was removed and a new anchor was used to anchor to fascia. It is important to mention that initial ITB catheter replacement required an L3-L4 laminectomy with the catheter anchored directly adjacent to the vertebrae. It is entirely unclear as to why laminectomy was needed for the ITP catheter revision as this was done at an outside facility with documentation not available to corroborate. A logical explanation was that he had this laminectomy performed after his acute spinal cord injury and not during the revision of the ITP catheter. These factors played a role in the undesired kinking of the catheter. As a result, in the final procedure, these concerns were addressed, anchoring the catheter to the supraspinous fascia, maintaining a substantial amount of space between the catheter and the surrounding vertebrae or bony prominences and interlaminar calcifications. However, it is important not to apply too much tension when securing the anchor to avoid the catheter snapping back while under strain. 

We focus on an underreported case of spasticity that is affected by the individual's position. Our patient's catheter might have become kinked due to frequently extending their spine. This would explain why spasticity was quickly alleviated when the spine was flexed. The following databases were used to determine whether there have been cases reported regarding ITB catheter occlusion as a result of the patient's position from 2000 to 2023: PubMed, Web of Science, Scopus, JSTOR, and Google Scholar. The keywords included position-dependent catheter occlusion, intrathecal catheter occlusion, indwelling catheter, intrathecal drug delivery. This is the first case report describing an intrathecal catheter being repeatedly compressed near the vertebrae, leading to intermittent baclofen withdrawal symptoms. Therefore, we suggest that there is a greater likelihood of spasticity recurring in patients who underwent a laminectomy for catheter placement, in addition to anchoring near the post-laminectomy site. The outcome prompts one to inquire about the normal appearance during the contrast dye study, since the kinking was sporadic and influenced by position. The catheter needed to be free of obstructions when injecting contrast, allowing the dye to flow without any hindrance. Looking back, it is possible that had we placed the patient in a prone position with rolls on their chest wall before the catheter dye study, we might have been able to identify the issue. 

One crucial point to consider in a clinical setting is the occurrence of occasional tolerance to baclofen, in which the physician may need to raise the dosage to address the spasticity. Due to the patient's complaints of spasticity, numerous dose changes were made prior to intraoperative exploration. Interestingly, the alternating periods of lethargy with flaccidity and spasticity further support the issue of being positional. This case demonstrates the importance of ruling out catheter pump malfunction as the cause of increased spasticity before attributing it to drug tolerance. 

Withdrawal syndrome from baclofen is a serious and potentially life-threatening situation that most commonly occurs after abrupt discontinuation of ITB therapy due to human error, ITB pump failure, intrathecal catheter migration, or after pump removal due to infection [15,16]. In such cases, additional oral or intrathecal baclofen should be administered immediately. Early symptoms include recurrence of baseline spasticity, fever, pruritus, or paresthesias. Persistent withdrawal can cause hallucinations, delirium, seizures, and muscle rigidity. Untreated abrupt ITB discontinuation can progress to rhabdomyolysis, severe autonomic instability, multiple organ failure, cardiac arrest, and death within one to three days. Severe baclofen withdrawal syndrome may resemble sepsis, malignant hyperthermia, autonomic dysreflexia, serotonin syndrome, neuroleptic malignant syndrome, or other hypermetabolic states with diffuse rhabdomyolysis [17]. 

It is crucial for a specialist or a trained caregiver to routinely examine the drug delivery system to minimize the risk of ITB withdrawal syndrome. Even in specialized centers, diagnosing malfunctions in the pump system can prove to be quite challenging. In our situation, a careful examination of the drug infusion device did not reveal any programming issues, and it was only through intraoperative exploration with replacement of the entire system that the problem was identified. 

The utilization of ITB pump delivery systems serves to effectively control medically untreatable spasticity. Nevertheless, complications with the ITB pump can arise from catheter obstruction due to improper placement, even though imaging may not show any issues. Several studies have explored ITB troubleshooting algorithms regarding the decrease in effectiveness of ITB therapy [18]. However, there is a lack of literature discussing the specific positioning that could make patients more susceptible to refractory spasticity caused by pump placement. It may be beneficial to use fluoroscopy to observe the inserted catheter during changes in the patient's position, particularly if there is a prior indication that symptoms are influenced by position. 

## Conclusions

We suggest that among the various troubleshooting techniques for a potential malfunctioning device, it is essential to consider the potential issue of a blocked catheter. This transient occlusion of the catheter resulted in a failure to administer ITB and led to a resurgence of his pain and spasticity which significantly improved following replacement. To decrease the likelihood of transient catheter obstruction, it is advisable to secure it firmly to the supraspinous fascia away from bony prominences. Additionally, this case report brings attention to those who are at a higher risk of catheter complications following a spinal cord injury. It is crucial to identify and promptly address this complication to prevent adverse side effects and potentially fatal drug withdrawals.
